# Influences of Crystal Anisotropy in Pharmaceutical Process Development

**DOI:** 10.1007/s11095-018-2374-9

**Published:** 2018-03-19

**Authors:** Eftychios Hadjittofis, Mark Antonin Isbell, Vikram Karde, Sophia Varghese, Chinmay Ghoroi, Jerry Y. Y. Heng

**Affiliations:** 1 Surfaces and Particle Engineering Laboratory (SPEL), Department of Chemical Engineering, Imperial College London, South Kensington Campus, London, SW7 2AZ UK; 2 DryProTech Laboratory, Chemical Engineering, Indian Institute of Technology Gandhinagar, Palaj, Gandhinagar, Gujarat 382355 India

**Keywords:** anisotropy, crystal engineering, crystal growth, dissolution, surface properties

## Abstract

Crystalline materials are of crucial importance to the pharmaceutical industry, as a large number of APIs are formulated in crystalline form, occasionally in the presence of crystalline excipients. Owing to their multifaceted character, crystals were found to have strongly anisotropic properties. In fact, anisotropic properties were found to be quite important for a number of processes including milling, granulation and tableting. An understanding of crystal anisotropy and an ability to control and predict crystal anisotropy are mostly subjects of interest for researchers. A number of studies dealing with the aforementioned phenomena are grounded on over-simplistic assumptions, neglecting key attributes of crystalline materials, most importantly the anisotropic nature of a number of their properties. Moreover, concepts such as the influence of interfacial phenomena in the behaviour of crystalline materials during their growth and *in vivo*, are still poorly understood. The review aims to address concepts from a molecular perspective, focusing on crystal growth and dissolution. It begins with a brief outline of fundamental concepts of intermolecular and interfacial phenomena. The second part discusses their relevance to the field of pharmaceutical crystal growth and dissolution. Particular emphasis is given to works dealing with mechanistic understandings of the influence of solvents and additives on crystal habit. Furthermore, comments and perspectives, highlighting future directions for the implementation of fundamental concepts of interfacial phenomena in the rational understanding of crystal growth and dissolution processes, have been provided.

## Introduction

Crystalline materials are ubiquitous in several sectors including electronics, cosmetics and pharmaceuticals, contributing significantly to the development of a wide range of products. Within the pharmaceutical industry, crystalline materials remain at the epicentre of most formulations. Understanding the processes underpinning the growth and dissolution of crystalline materials is therefore of crucial importance in the development of more efficient drug formulations.

Crystals are multifaceted entities, with each facet carrying different properties depending on the orientation of the molecules in the crystal lattice. This leads to the concept of crystal anisotropy, which holds true for several properties of crystalline materials, including optical, magnetic, and surface properties. The concept of anisotropy was extensively studied in the electronics industry towards the late nineteenth century (1,2), notably for semi-conductors. For crystalline pharmaceutical solids, experimental evidence of facet specific properties has also been established (3–7). Nevertheless, the importance of crystal anisotropy in pharmaceutical process development remains omitted, mainly owing to the lack of a sufficiently developed framework for the implementation of anisotropic properties in mechanistic modelling of pharmaceutical processes. The emphasis of this review is on the importance of crystal anisotropy in crystal growth and dissolution, two processes of major concern for pharmaceutical industry. Especially for the former, it is critical to understand the importance of controlling crystal habit during crystallization; crystal anisotropy is critical in processes downstream of crystallization (8–10). Obtaining a desired crystal habit upstream could potentially allow the optimization or even elimination of downstream operations such as milling and granulation. These processes modify the crystal habit, but their output heavily relies on the crystal habit of the input material. Thus, understanding the rise of different crystal habits during crystallization is definitely not a subject of strict academic interest. It is a topic that could, potentially, transform the design of pharmaceutical processes.

This review is aimed at an audience with little to no exposure in the development of rational crystal engineering approaches for the design and production of particles with tailored properties. It begins with an overview of the aforementioned theoretical concepts associated with the performance of pharmaceutical crystals, with an emphasis on surface energy anisotropy. Following that, the importance of the latter in crystal growth is discussed. Numerous theoretical and computational studies, highlighting the importance of anisotropic surface interactions in the determination of crystal habits, are introduced. The interactions of different crystal facets with solvents and additives are examined, and experimental studies are presented. The review, also, discusses several concepts related to crystal dissolution. Particular emphasis is given on topics such as crystal polymorphism, the importance of surface energy anisotropy and the influence of surface coatings. Both modelling and experimental approaches are reviewed.

The authors also provide their own perspectives on the field following the review. They highlight the differences between crystal growth and dissolution, two processes which are not reversible nor opposites. Furthermore the need for a thorough understanding of the dissolution and growth media, which includes all the components of surface energy including the importance of surface active agents, is discussed.

## Concepts in Crystal Growth and Dissolution

### Polymorphism

The ability of the constituent components (atoms, ions or molecules) of a solid substance to arrange themselves to more than one crystalline phases is defined as polymorphism. This concept was officially introduced by Professor Eilhard Mitscherlich (11) in 1826, when he presented evidence for the existence of monoclinic and rhombic crystals of sulphur. Based on his results he called this phenomenon dimorphism. Earlier, in 1825, he reported, for the first time, experimental proof for the different physical and structural properties of different polymorphs. Polymorphs of calcium carbonate were used in this study. Intriguingly, in these studies Mitscherlich identified, probably for the first time, scientific evidence for the anisotropic nature of crystals, reporting different rates of expansion/contraction of different crystal facets.

**Fig. 1:**
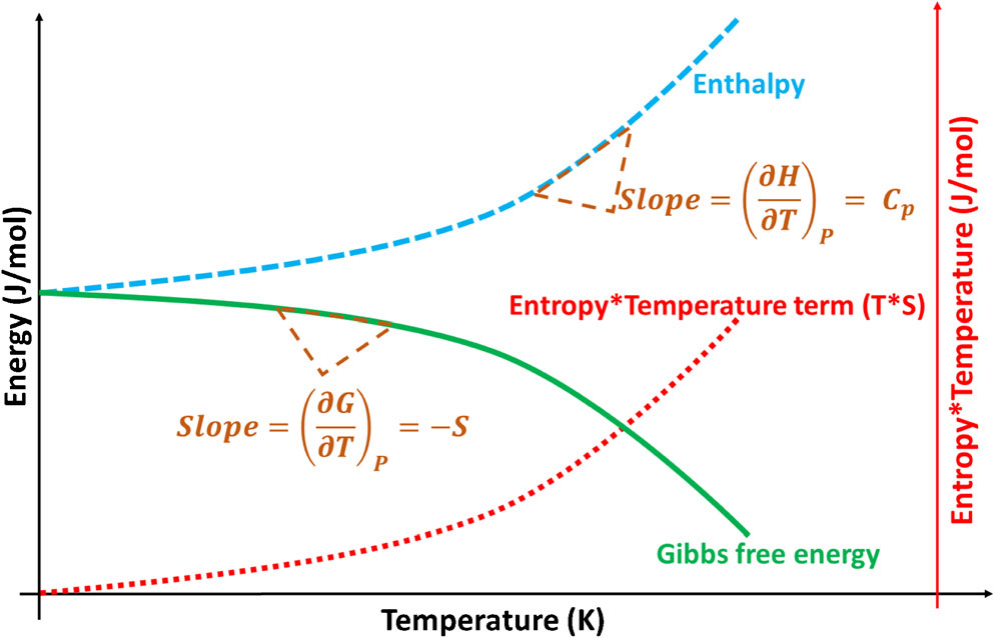
Diagram showing the variation of Gibbs free energy, enthalpy, and entropy with temperature for a crystalline solid material. The slope of the enthalpy curve gives the value of heat capacity and the value of the Gibbs free energy slope gives the value of entropy. For pharmaceuticals, the importance of pressure is usually omitted, since the operations are performed in conditions where the influence of pressure is considered negligible

From a thermodynamic standpoint, for a certain compound at a set paired temperature and pressure, there is only one stable polymorph. The rest of the polymorphs are considered metastable, and tend to transform to the stable form by means of a solid-state transformation. The Gibbs free energy of a solid, as depicted in Fig. 1, is the thermodynamic parameter used to assess the stability of each polymorph and is calculated, for a polymorph *i*, according to the equation:

1 $$ {G}_i={H}_i-{TS}_i $$

**Fig. 2:**
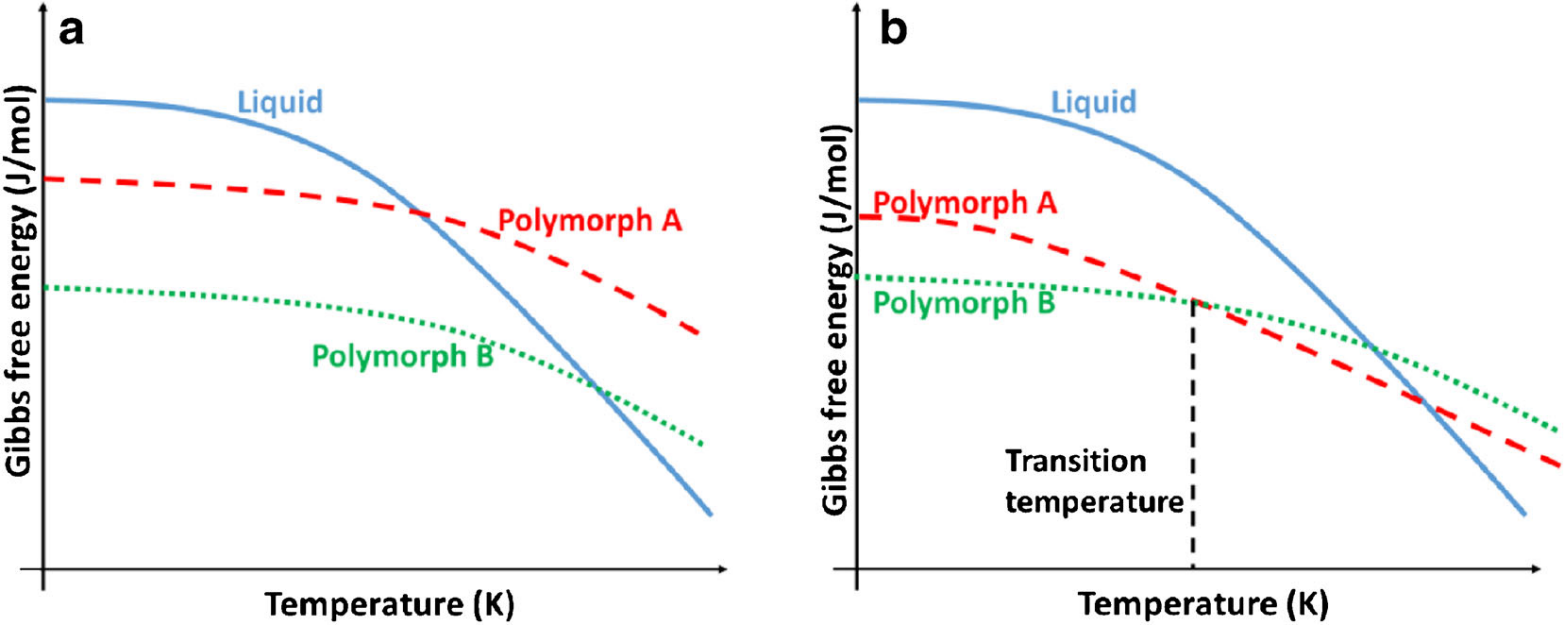
The Gibbs free energy diagrams for (**a**) monotropic and (**b**) enantiotropic conditions. For the enantiotropic situation the transition temperature is also marked. In the monotropic case, polymorph B is the stable polymorph, where in the enantiotropic case, polymorph B is the stable polymorph initially and polymorph A is the stable one beyond the transition temperature

As the conditions change, a shift in the relative magnitude of the value of the Gibbs free energy of two polymorphs can occur, leading to a change in the order of stability called enantiotropic behaviour. On the other hand, some solids exhibit only a single stable polymorph which is called monotropic behaviour. In this case all the other polymorphs appear as metastable. These phenomena are illustrated in Fig. 2.

Owing to the kinetic nature of crystallization, certain polymorphs can be exhibited at temperatures where they are considered metastable. Thus, the system would start to exhibit a behaviour associated with the Ostwald rule of stages, applied to the context of crystallization (12) by the following excerpt from the original body of work (13):

“At a sufficiently high supersaturation the first form that crystallizes is the most soluble form. This transient state then transforms to the more stable form through a process of dissolution and crystallization.”

In other words, since the activation barrier for the metastable polymorph is lower, the higher rates of the reaction may favour the nucleation of the metastable polymorph. The metastable polymorph would then tend to transform to the most stable form over time.

A vast array of experimental techniques are currently implemented for the identification of polymorphs in the pharmaceutical industry. Polarised optical microscopy (14), differential scanning calorimetry (DSC) (15), X-ray diffractometry (XRD) (16), infrared (IR) and Raman spectroscopy (17,18), inverse gas chromatography (IGC) (19), and nuclear magnetic resonance (NMR) (16) are just some of the techniques used in polymorph identification. Despite all of the tools offered, quantifying polymorphic content is not always straightforward, and often requires complex computational algorithms.

Carbamazepine and mefenamic acid are two clear examples, highlighting the limitations of some of the aforementioned techniques. For instance, it is well reported that crystals of mefenamic acid form I, crystallized from a supersaturated toluene solution (20) are needle shaped, similar to crystals of mefenamic acid form II (21). Similarly carbamazepine polymorphs II, III and IV have been isolated as needle shaped crystals as well (22). The occurrence of crystals of the same molecule, but of different polymorphic form, with the same crystal habit poses limitations for the identification of the different polymorphs by means of optical microscopy. Additionally, the study of carbamazepine’s polymorphism exposes the limits of both DSC and FTIR as means of polymorph identification. For instance, the melting temperatures of polymorphs I, II and III are 193.5, 192.1 and 193.2°C respectively; and all three polymorphs have very similar FTIR spectra. In this context, it is not a coincidence that XRD is seen as the most reliable technique for polymorph identification.

Computational tools have been used extensively for the prediction of the different polymorphic forms (23–25). These tools are predicting a large number of polymorphic structures, though the existence of some of these predicted structures has not been confirmed experimentally as of yet. These simulation studies showcase the importance of different parameters (conformational flexibility, functional groups, etc.) in the determination of the free energy of a crystal lattice. Given the framework determining polymorphism, the next step is to understand how different species manifest themselves at a macroscopic level in the form of crystals. The first step is to understand how crystal growth from first principles.

### Defects in Crystalline Materials

In a footnote in his pioneering essay on the “Equilibrium of Heterogeneous Substance”, published in 1878, Professor J.W. Gibbs (26) made the following statement highlighting the possibility for the growth of perfect crystals (where the term ‘perfect’ refers to crystals where their only defects are their surfaces):

“Single molecules or small groups of molecules may indeed attach themselves to the side of the crystal but they will speedily be dislodged, and if any molecules are thrown out from the middle of a surface, these deficiencies will also soon be made good; nor will the frequency of these occurrences be such as greatly to affect the general smoothness of the surfaces, except near the edges where the surfaces fall off somewhat, as before described. Now a continued growth on any side of a crystal is impossible unless new layers can be formed.”

A number of pioneers on crystal growth has developed theoretical approaches for the growth of perfect crystals, via the nucleation of two dimensional layers. However, it was soon realised that in the supersaturation range where crystals usually grow, two dimensional nucleation is not easily achievable. An extensive series of studies conducted by researchers such as Frenkel, Burton, Cabrera, and Frank, revealed the importance of topographical defects in crystal growth, suggesting that these features are necessary for a more coherent explanation of crystal growth at low supersaturations.

As mentioned, crystal defects are an inherent part of crystal growth, but they can also be induced on crystals, by thermal or mechanical stresses. Generally speaking, they are characterised as having a higher free energy than the bulk of the material and thus are preferential reaction sites (either via Lifshitz-van der Waals or chemical interactions).

Defects are distinguished into three large categories on the basis of their nature:

1. Point defects: This case includes situations where atoms or molecules are missing from a point on the crystal lattice creating a vacant point, or when an extra atom appears at a random position on the lattice.
2. Line defects: This is when a group of atoms/molecules is misplaced in the lattice creating screw or edge dislocations.
3. Plane defects: In this case defects appear in the region between two homogeneous crystal planes.

Atomic force microscopy (AFM) is, usually, employed for the study of defects in crystalline materials. The technique allows for very accurate studies, even though the nature of the experiment means the AFM tip may affect the sample being investigated, both in dry or wet conditions. Adsorption based techniques, such as IGC, appear to have the potential to provide quantitative data on the number of surface defects present in crystalline materials, along with the surface energies associated with them (27). Such defects are the basis from which all crystal growth occurs and allow us to better understand how facets of crystals are exhibited. These facets can be best studied and understood through their changing surface energies.

### Surface Energy

**Fig. 3:**
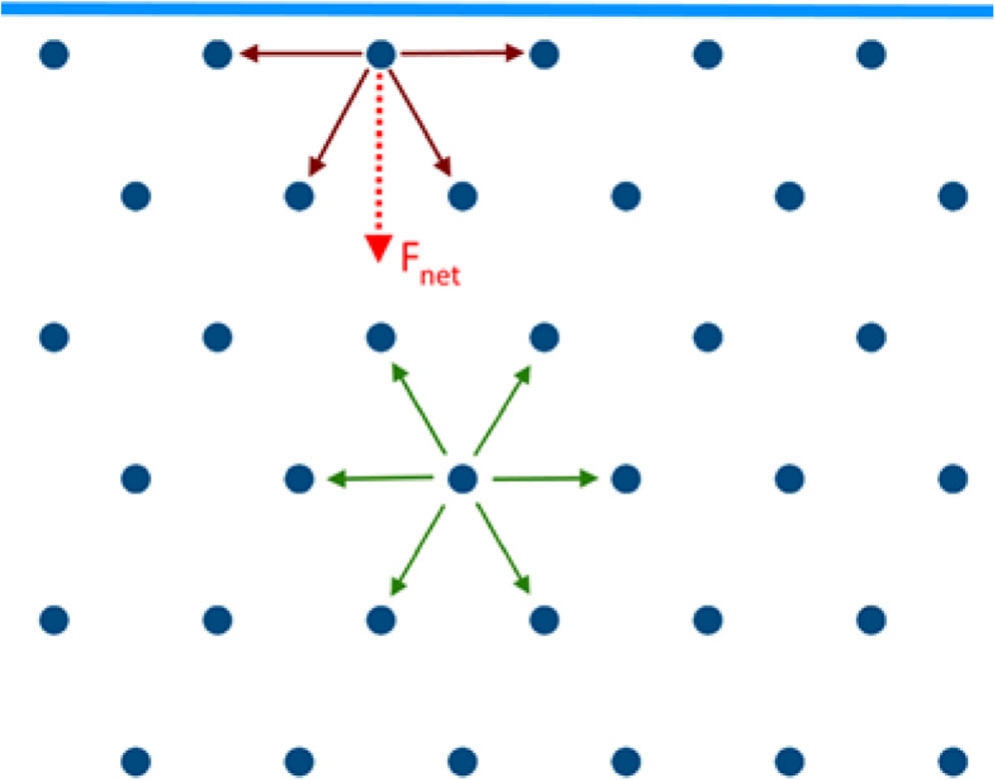
Schematic for the anisotropic nature of intermolecular bonding between an interface and the bulk giving a surface free energy

Surface energy (γ) arises from the anisotropic interactions at the surface of a substance, as shown in Fig. 3. Considering the range of functional groups and the associated types of interactions, surface energy has been proposed to be deconvoluted (28,29) in two major components, a Lifshitz-van der Waals (γ^LW^) component and an acid-base (γ^AB^) component. The former term includes London (30,31), Debye and Keesom interactions. On the other hand, the acid-base component can be further deconvoluted to an acid (γ^−^) and a base (γ^+^) component. This analysis, developed by van Oss, Chaudhury and Good, has been named after the initials of its founders as vOCG (32) and proposes a geometric mean relation between the acid and the base components. Its mathematical formulation is given as follows:

2 $$ \gamma ={\gamma}^{LW}+{\gamma}^{AB}={\gamma}^{LW}+2\sqrt{\gamma^{+}{\gamma}^{-}} $$

On the same grounds, the work of adhesion between two surfaces, here 1 and 2, is described by the following equation:

3 $$ {W}_{12}={2}^{\ast}\left(\sqrt{\gamma_1^{LW}{\gamma}_2^{LW}}+\sqrt{\gamma_1^{+}{\gamma}_2^{-}}+\sqrt{\gamma_1^{-}{\gamma}_2^{+}}\right) $$

One should note that if the two surfaces are identical, the term work of adhesion becomes work of cohesion.

Despite the fact that the work of adhesion is usually considered to be reversible, meaning that it’s equal in absolute magnitude to the work of separation between the two objects, this has not been verified experimentally. In fact, measurements (33–38) performed both on solid-solid and the solid-liquid interfaces reveal that a number of different factors contributing to an inherent irreversibility of the process of adhesion-separation; meaning that it is not the experimental procedure that leads to the irreversible behaviour, but the nature of the process per se.

The concept of the Lifshitz-van der Waals component of surface energy is quite well established. However, the exact nature of the acid-base component is a field of active discussion. The geometric mean approximation, used to describe acid-base interactions, does not have a solid theoretical background and it has been adopted on the basis that it has a theoretical meaning for the Lifshitz-van der Waals component. The harmonic mean approximation has been employed instead of the geometric mean one; however, there is no theoretical explanation for why acid-base interactions can be modelled using the harmonic mean approximation.

Each crystal facet possesses different surface energy values. This means that all the processes associated with surface energy, including crystal growth and dissolution, are facet specific. Therefore, a mechanistic understanding of these processes requires an understanding of the facet specific surface energy. Depending on the nature of the material of interest, different experimental techniques can be used to measure the surface energy of solids. All of them use a number of different solvents to measure the interactions between the solid and the particular solvents. Then, the different models, proposed in literature, were used to calculate the values of surface energy, or the acid-base work of adhesion.

In the past ten years, a number of investigators have managed to grow macroscopic crystals of different compounds of pharmaceutical interest and calculate the surface energies of individual facets using contact angle goniometry. X-ray photoelectron spectroscopy has been used as a complimentary technique to confirm anisotropic surface properties. More recently IGC measurements and computational models for deconvolution have been developed enabling the link between contact angle and IGC measurements (39–41). Having understood how we measure and study surface energy, we can now see how this is reflected in exhibited crystalline facets specifically, otherwise known as crystal habits.

### Crystal Habit

The fundamentals behind crystal growth, surface energy, and the parameters that govern them were discussed in the previous section. Crystal habit refers to the shape of the crystal, determined by the facets present*.* Here, the concept of crystal habit, or in other words, the facets that are exhibited macroscopically are discussed. Crystalline facets are related to unit cell lattices and are usually assigned using Miller’s Indices, based on the following notation (*hkl*). They are numbered relative to their orientation in the crystalline plane as integers such as [100], [010], [102]. As mentioned earlier, control of crystal habit during crystal growth and the associated anisotropy could play a crucial role in guiding pharmaceutically relevant processes or unit operations like dissolution, milling, compaction, flow and granulation. Hence, both the thermodynamic and mechanical aspects allied to the anisotropy in crystalline materials should be given diligent attention during pharmaceutical formulation and development process.

As with a lot of the work in this field, Gibbs would be the first to formally approach this topic (26); ultimately introducing the crucial idea that, thermodynamically, the crystal habit should seek to minimize total surface energy in anisotropic particles. The minimization of the total surface free energy of the crystal-medium interface, is calculated as the sum of the surface energies (γ) by surface area (A), for each facet exhibited (*i* or *hkl*). Wulff, and later Chernov, would suggest that each facet quantity was proportional to a vector normal to the facet from the centre of the crystal, called the Wulff-Chernov construction (42). Thus crystal habits are dictated by the growth rates of particular facets, with the slow growing ones being more exhibited at the surface.

Several models were later proposed to predict crystalline habits, with the two below being considered the original classical ones:

1. The first true predictive approach would come from the Bravais-Friedel-Donnay-Harker (BFDH) model (43), founded upon the Bravais relationship where the perpendicular growth rate of a facet (*R*_*hkl*_) is inversely proportional to its interplanar spacing (*d*_*hkl*_). The model is purely crystallographic in its nature and therefore the simplest given the limited amount of data required to make estimates.

4 $$ {R}_{hkl}\propto {d}_{hkl} $$

2. The next model is from Hartman & Perdok (44) who claimed that the faster the growth rate was, the higher the bond energy of that facet. Similar to the model above, the perpendicular growth rate is therefore predicted to be proportional to the bond attachment energy, i.e. the bond energy released for each molecule becoming a part of the surface (${E}_{hkl}^{AE}$).

5 $$ {R}_{hkl}\propto {E}_{hkl}^{AE} $$

Bond attachment energy (${E}_{hkl}^{AE}$) is separate from the surface energy (*γ*_*hkl*_) of a facet. The former is specifically the energy released per molecule when a uniform molecular layer with interplanar spacing (*d*_*hkl*_) bonds to the facet. As evident from the definition it is specific to layered growth, unlike surface energy which lacks this molecular directionality, related to crystallization.

Both of them lack any microscopic understanding of the growth mechanisms and do not account for any hydrodynamic phenomena, and have been known to give unreliable predictions at times. Therefore more mechanistic models, accommodating a more molecular approach, have been developed, notably with the furthered understanding of the important role of surface defects discussed in the next section, dealing with crystal growth. Having gone through the fundamentals of crystalline anisotropy, we can now look to see how this was observed at the molecular level; in other words the actual formation of such crystals.

### Crystal Growth

**Fig. 4:**
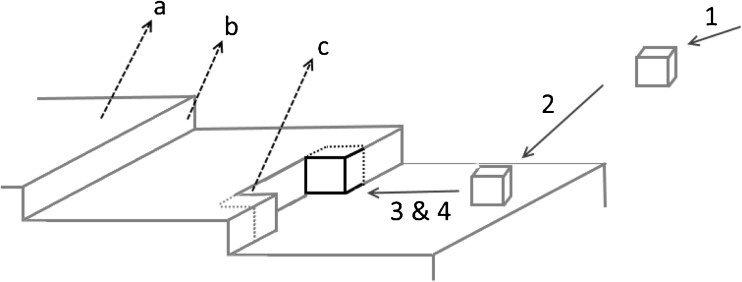
A schematic of crystal growth using the Kossel model. The numbering signifies the steps undertaken by the molecule to move from the bulk (1), to diffuse to the solid surface (2), for it along with the surface to dessolvate (3), and finally for it to be incorporated into the solid shown with the black outline (4). The lettering represents the topographical features: a. the terrace, b. the step, and c. the kink site of preferred attachment

Crystal growth, in liquid, is the step following nucleation where a crystalline solid will form and continue to grow until equilibrium is achieved with the surrounding liquid phase (45). This only occurs if the free energy of the molecules in the solid is lower than that of those in the solution. This step involves the addition of solute molecules onto the crystal surface and becoming a part of the lattice. Chronologically it typically involves the following four steps, typically called Kossel model, illustrated in Fig. 4:

1. The bulk transport of the solute to the facet
2. The surface diffusion of the solute on the solid surface across the crystal facet
3. The desolvation of both the site and the solute molecule
4. The bond attachment of the molecule onto the facet

One of the first points understood on crystal growth is that surface diffusion is typically the rate limiting step during growth, in the range of supersaturations usually employed. Furthermore, it was shown that the attachment frequency of a molecule onto a perfectly flat surface would be very slow and by extension so would the growth process. Once a molecule was attached, it would form the defects that would greatly diminish the energetic cost and so speed growth. These preferred attachment points are the defects mentioned earlier in the review, and are shown and described as kink sites in the Fig. 4. Though this process of layered growth, 2D nucleation, has a low likelihood if the degree supersaturation is not high enough, as mentioned in the section on defects in crystalline materials (45–47); however experimental data showed that growth would occur despite low degrees of supersaturation.

This understanding allowed researchers such as Frenkel (48) to realize that monolayers will naturally become rough and therefore seek to maximize the number of such kinks. One major mechanism for the formation of such sites is spiral growth where a plane dislocation forms a step that will fully regenerate. This mechanism was utilized by Burton, Carbrera, and Frank in their BCF model (46,49). The concept of intrinsic defects, or that crystalline materials are not perfect, allowed them to better predict crystal growth of various facets. They determined the perpendicular growth rate to be equal to the height of the step (*h*_*s*_), multiplied with the step velocity (*v*), and lastly the interstep distance (*y*). All three of these parameters are variable depending on the conditions imposed. The BCF model links crystal growth to the crystalline habit.

6 $$ {R}_{hkl}=\raisebox{1ex}{${h}_sv$}\!\left/ \!\raisebox{-1ex}{$y$}\right. $$

The BCF model has been refined by several researchers, most notably by Chernov who moved away from the Kossel crystal lattice assumption (45). The Kossel model, used in Fig. 4, assumes all monomers to be cubic blocks and isotropic, and therefore removes most molecular distinctions.

One notable development within this model was the observation of a critical length (*L*_*c*_) for the step. It was experimentally determined that for a step to flow outwards (parallel to the facet), it had to be of a certain length. Without this, the spiral mechanism would not take place as a new edge would not be present. This is notably seen in the works of Rimer and Vekilov (50–52), who showed that it was related to the chemical potential difference (*∆μ*) which provides a measurement for the supersaturation, the liquid-solid interfacial tension (*γ*), and the volume occupied by one molecule in a crystal (*ω*). The inclusion of chemical potential difference and the liquid-solid interfacial tension means that a wide variety factors are subsumed under them. These include but are not limited to supersaturation, viscosity, solvent, temperature, which gives this model a tremendous amount of flexibility.

7 $$ {L}_c=\raisebox{1ex}{$\gamma \omega $}\!\left/ \!\raisebox{-1ex}{$\Delta \mu $}\right. $$

An alternative take on the crystal growth modelling problem focusing on kinetics and the solid-liquid interface was pioneered by both Boek and Bennema (45,49,53). The BCF model was modified to include the diffusion of the solute molecules from the solution to the kinks. Furthermore, on the same work the authors incorporated on the BCF model the concepts of desolvation for the solute to become incorporated at the solid interface, in Fig. 4 it is the 3rd step. Effectively Boek and Bennema split the activation free energy ($\Delta  {G}_{hkl}^{\ast }$) into two steps, the desolvation and the incorporation into the kink. Ultimately the concept of molecule attachment frequency was introduced, to account for the time required for molecules to appropriately orient themselves prior to be adsorbed, becoming a part of the lattices.

Further developments still, have revolved around molecular simulations with the work by Gilmer and Bennema (54) using a Monte Carlo simulation being one of the first. Good agreement was found with the known mechanistic rationales of the time, though only at set conditions notably at low supersaturations. Later improvements have come from Molecular Dynamics, notably from Boek (55–57), investigating the influence of the water shell surrounding the urea molecule. This work by Boek showed that the arrangement of water molecules depended in part on the facet exhibited by the crystal at the interface, reaffirming the need for a strong molecular mechanistic rationale (58).The above sections were a dense review of the fundamentals of crystalline anisotropy, how they are observed, studied and formed. Within the context of pharmaceuticals, their importance lies in how they will be consumed by the patients, their dissolution performances. This next section deals with these fundamentals and seeks to connect the growth of anisotropic crystals to their dissolution.

### Thermodynamic Basis for Dissolution Processes

Dissolution performance of a dosage form is a critical quality control criterion in the pharmaceutical industry (59,60). Typically, the in-vitro dissolution performance is used as a surrogate for *in vivo* bioequivalence testing. In general, the different sequential steps involved in the dissolution of orally administered pharmaceutical solid dosage form follows the wetting of the solid surface by the liquid, with the subsequent disintegration and deaggregation of the solids in the medium (solvation, referred to as a surface reaction), and finally the diffusion of the solute into the bulk fluid. Thus, the solvation of the drug molecules is a pre-requisite to the dissolution process into the bulk solution.

Dissolution processes are governed by the second law of thermodynamics. Dissolution results in the disruption of intermolecular bonding between the solute molecules and forms new solute-solvent interactions causing an increase in the degree of disorder. The mass transfer of drug molecules into the dissolution medium occurs by diffusion and convection mechanisms, depending on the features of the system. Diffusive mass transfer takes place due to the chemical potential gradients of the molecular species, and the random thermal motion of the molecules. In other words, the mass transfer is said to be dependent on the concentration gradient. The concept of drug dissolution is generally examined in the context set by the regulatory agencies of each country. For example, for the United States Pharmacopoeia (USP), one could look to chapters <711>, <724>, <1088>, <1090> and <1092> (61). Owing to the nature of *in vivo* dissolution process, diffusion based models are usually employed by regulatory bodies in order to assess drug solubility. In addition, it is worth noting that convective phenomena are also involved in dissolution (62,63).

In 1855, Adolf Fick (64), with his intuitive analogy between the molecular diffusion and conduction of heat and electricity, provided a fundamental equation to describe the diffusion mechanism which would be later known as Fick’s law of diffusion. Fick’s first law of diffusion states that, the rate of mass transfer (J) through a unit area (A) in a direction (x) is directly proportional to the concentration gradient (dC/dx), as shown here:

8 $$ \mathrm{J}=-D\kern0.75em dC/ dx $$

Where, J is mass transfer per unit area, also known as flux, C stands for the concentration, and D is the diffusion coefficient, a property which depends on the solute, fluid medium, and temperature of the system. It is important to understand that as the dissolution proceeds, the amount of solute in the bulk liquid increases and thus the driving force for dissolution and mass flux decreases. Despite the fact that in vivo dissolution is goverened by a combination of convection and diffusion mechanisms, Fick’s first law remains the most popular in predicting the dissolution rate and process in pharmaceutical systems.

In 1897, Noyes –Whitney (65) provided an empirical equation for the dissolution of a planar surface of constant surface area exhibiting Fick’s diffusion mechanism. It was proposed that, at constant surface area of a dissolving solid, the rate of dissolution (dQ/dt) is directly proportional to the solubility (C_s_) and bulk concentration (C_b_). Furthermore, for soluble solids this process can be considered zero order if the bulk concentration does not change.

9 $$ \frac{dQ}{dt}=k\ \left({C}_s-{C}_b\right) $$

Here k is the dissolution rate constant (mass transfer coefficient). This model is simplistic and does not provide a physical meaning for the rate constant ‘k’. Subsequently, a more sophisticated version of the model was given by Nernst–Brunner (66) which proposed that rapid equilibrium occurs initially at the solid-liquid interface followed by the formation of a stagnant hydrodynamic diffusion layer. The dissolution rate then depends on the mass transfer through the diffusion layer into the bulk solution. The dissolution rate constant (k) could now be related to the thickness of the diffusion layer (h), the diffusion coefficient (D) in the layer, and the surface area (A), assuming that the concentration gradient across the diffusion layer was constant. Thus, according to Nernst-Brunner model the dissolution rate (dQ/dt) is given by the concentration gradient from the surface of the exposed solid, and the diffusivity across a hydrodynamic boundary layer of thickness (h).

10 $$ \frac{dQ}{dt}= DA\frac{\left({C}_s-{C}_b\right)}{h} $$

Such transport controlled dissolution models are presumed superior and are very popular amongst pharmaceutical scientists. Based on it, Higuchi proposed a model which included the rate of release of solute from a matrix tablet (67). This model considers the drug dispersed in solid matrix and for planar geometry, is given by the corresponding equation:

11 $$ M=A{\left[D\ {C}_s\left(2{C}_0-{C}_s\right)t\right]}^{1/2} $$

Where, M is the total mass released in time t and C_0_ is the initial drug loading. This model was later extended to other geometries and porous systems (68).

### Predictive Modelling for Dissolution

To improve pharmaceutical drug design and development, substantial efforts have been expended towards modelling and predicting wetting, solubility, and dissolution behaviours. To elucidate and predict wettability and liquid-solid phase interactions during dissolution, MD simulations, involving classical force-field approximations, like statistical associating fluid theory (SAFT), have been used in the past couple of decades (69,70). Recently, tools like COSMO-RS resulting from the efforts of Klamt and co-workers, have gained considerable attention for the prediction of different thermodynamic and physiochemical properties, including wettability and solubility of small to medium sized molecules (71). Further improvements, in the form of the COSMO*frag* method, now make it possible to screen large numbers of potential molecules for drug design applications (72).

**Fig. 5:**
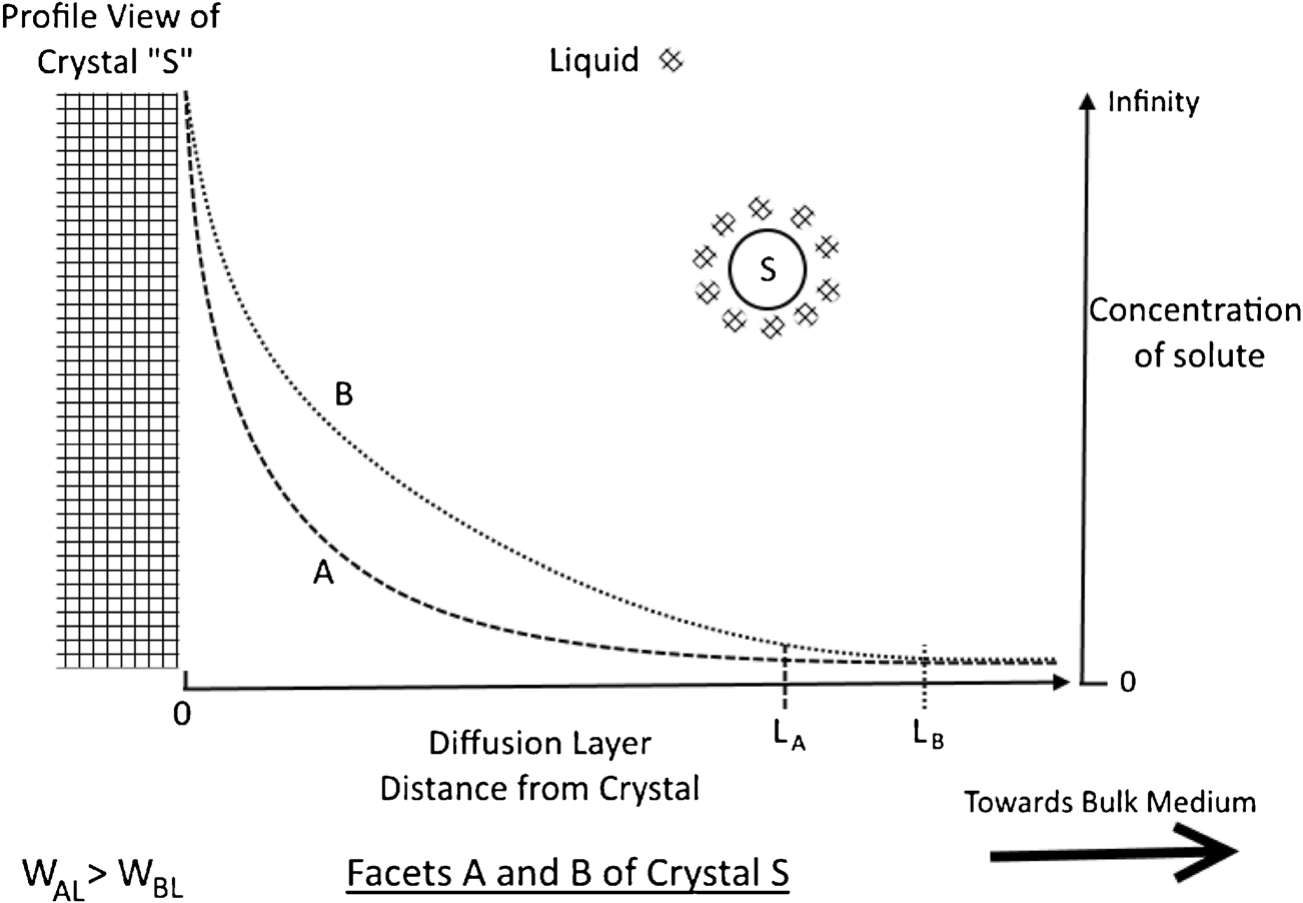
Schematic depicting the diffusion layer for two facets A and B of crystal S. From the legend it can be seen that the work of adhesion between facet A and the bulk liquid is bigger than the work of adhesion between facet B and the bulk liquid

In addition to simulating the dissolution process, researchers have now started to look a step further, by predicting the dissolution and oral absorption of drugs through mathematical models incorporating both dissolution and precipitation theories (73,74). These models could address the complexity in predicting the bioavailability of various drugs by describing complex kinetic dissolution-precipitation behaviour through physiological based modelling. Eventually, the introduction of anisotropy in crystalline solids and the resulting complex dissolution behaviours, to these theoretical and computational models, could be the next step towards a more accurate description and prediction of the dissolution process in crystalline materials. Figure 5 is a qualitative figure which shows how greater wettability of a facet favours its dissolution.

## Literature Review

### A Brief Overview of Current Research into Nucleation

Prior to crystal growth, nucleation must occur to give rise to a solid from the previous all liquid system. In this review, we do not approach the topic in depth, but it is crucial in the development of a mechanistic description of crystallization and in understanding which polymorphs are manifested (75,76). The formation of this liquid-solid interface is not energetically spontaneous and is affected by many of the same factors that affect crystal growth (77). Nuclei, in the classical sense, are defined by a critical diameter below which it is energetically unfavourable and wants to dissolve, and above which the nucleus will spontaneously grow. In other words, a nucleus on its critical size is just as likely to dissolve as to grow.

Nucleation was first modelled according to classical nucleation theory. While the concepts behind it, including a thermodynamic critical size, are still useful in helping us understand the phenomenon, there are severe limitations to it. Notably, the theory was found inadequate to describe crystallization kinetics, and therefore changes and even newer equations have been adapted to more accurately predict nucleation. Vekilov *et al*. introduced a phenomenological model which suggested an intermediate step in nucleation (78). A highly dense metastable pre-nucleation liquid droplet within which nuclei would form, was proposed. The model was reliable for proteins and corroborated with experimentally determined nucleation rates. Other academics such as Gebauer *et al*. and Sleutel *et al*. have offered evidence to support the presence of such pre-nucleation clusters for alternative systems to proteins (79,80). However, computational molecular simulations from Zahn and structural sampling techniques from Parinello suggest that such clusters may actually impede nucleation in certain cases (81,82). Other groups, such as Yoreo and Rimer *et al*., have offered an alternative approach following particle attachment theory. Their body of work has been to subsume various theories into an encompassing branch of particle attachment, including the pre-nucleation clusters, though their work was developed from crystal growth. Another major advancement in modelling nucleation has focused on the effect of diffusive interfaces in the phase transformation, first presented by Cahn *et al*. (83). It remains unclear, whether the solution conditions influence the crystal anisotropy of the fundamental crystalline particle. This means that despite the advancements, the mechanism dictating the rise of a faceted entity from an amorphous one is still unknown.

### The Influence of Solvent in Crystal Growth

Snowflakes were probably the first system for which crystal habit was extensively investigated as a function of different parameters. From the early seventeenth century, scientists were already investigating their crystal habits, but it was not until 1932 that a systematic study was performed. At that time, Professor Ukichiro Nakaya (84) started examining the crystal habit of snowflakes produced at different temperatures and different values of water vapour saturation. His results were tabulated in the Nakaya diagram.

In the field of industrial crystallization, the crystal habit of urea (85) was one of the first to be studied. A publication in 1936 documented that the urea produced in the USA, crystallized in alcohol, was rhombus like, contrary to the needle shaped imported urea, crystallized in water. The rhombohedral crystal habit offered a great advantage in terms of flowability. Using arguments similar to those outlined in the crystal habit section of the theory section, one can claim that certain facets interact favourably with water molecules, slowing down their growth rates. Thus, these facets dominate compared to the faster growing facets. In the case of water, the rate of growth of each facet, described by this simple formalism, is such that it gives rise to needle shaped crystals.

The first attempts to understand this behaviour were done with the aid of a mechanistic model based on the Burton-Cabrera-Frank approach. The model was parameterized using the results from force field simulations and it was able to capture the needle shaped structure of urea crystals grown in water and the cuboid habit of crystals grown in polar solvents (86). A similar model was successfully implemented for the study of amino acids (87).

The breakthrough in the field was achieved with the implementation of the concept of the solid-liquid interfacial interactions in the modelling methodology, as referenced in the crystal growth section earlier. Molecular dynamic (MD) (56,57) simulations were performed on individual facets with respect to different solvents, enabling the development of a library containing all the necessary information. These were later fed into a model, based on the Wulff-Chernov (42) formalism, to construct diagrams accurately predicting the crystal habit for different solvents at different supersaturations (88). Even though the process employed for the prediction of the crystal habit of urea offers accurate results, it has many bottlenecks; the most notable being the length of time required for MD simulations. This makes it difficult to implement the influence of additives in the system, as simulations including molecular additives would significantly increase the computation time.

The understanding of the rate of attachment/detachment (89–92) on kink sites, was accompanied by the development of formalisms on the study of spiral growth on individual facets, and on the influence of solvents (93–95). The advancements enabled by the development of mechanistic models for the prediction of the crystal growth. In these studies, the concept of periodic bond chains, introduced by Hartman and Perdock (96), was used in order to describe the anisotropic rates of attachment and detachment associated with the spiral growth. It was shown that during the spiral growth process, the most time consuming part for each revolution was the lengthening of the edges. AFM images verified that the spiral structure, predicted by computational models, were expressed on the crystal facets.

The efforts, towards an integrated computational framework for the prediction of crystal habit, culminated in the development of the ADDICT algorithm. ADDICT was tested and found to successfully capture the crystal habits of molecules of pharmaceutical interest such as acetaminophen, lovastatin, δ-mannitol, and glycine in a number of solvents (97).

These mechanistic approaches, appear to be versatile tools for the determination of crystal habit under various conditions. In the latest versions, the surface energies of both the solvent and the solid surface are a key component of the algorithm. These models have several assumptions, the most notable one being that in the absence of hydrogen bonds, the whole acid-base component of the surface energy is assumed to be zero. However, given the empirical character of the geometric mean approximation for the acid-base interactions, doubts arise about the range of results that can be obtained, suggesting areas of improvement. In addition, certain aspects of the behaviour of fluids at interfaces could be implemented (98). This could include the formation of “clathrate cages” by water molecules around hydrophobic molecules where the average number of hydrogen bonds is higher than in the bulk. Finally, an experimental validation of the proposed values for both the surface energies of individual facets, as well as their corresponding work of adhesions with different solvents, could provide guidelines for the enhancement of the currently existing methodology.

Shah *et al*. (20) used mefenamic acid form I as a model compound to experimentally study the influence of solvent on crystal habit. Using a range of solvents, it was shown that mefenamic acid can crystallize in a broad spectrum of crystals, ranging from needles to thick plates. In this study, it was possible to correlate the aspect ratio of the crystals with the polar component of the solubility parameter of the solvent. The work did not investigate the influence of degree of supersaturation, which may affect the interfacial properties.

More detailed studies (99) were performed on the crystal habit of acetaminophen for solvents with different polarities. For polar protic solvents, it was found that their affinity towards facets favouring hydrogen bonding slowed the growth rate of those facets, since the sites were less prone to interact with solute molecules. For polar aprotic solvents, where hydrogen bonding was weaker, there was more ambiguous relationship. These results highlight that simulation tools and/or complimentary techniques (such as XPS) are paramount for understanding crystal growth.

The above findings complimented studies (100,101) performed on carbamazepine dihydrate, a hydrophobic channel hydrate, which usually crystallizes as needles. Investigations on the structure of the compound suggest that the elongation is driven by the formation of a hydrogen bond network, owing to the presence of water in the crystal lattice. Thus, in this case, hydrogen bonding drives the crystal growth in one axis. This easily explains why the crystals grow into needles in pure water; though aqueous alcohol mixtures are more often used to crystallize carbamazepine dihydrate. It has been observed, that the more protic the alcohol is, the higher the aspect ratio of the crystals.

Attempts have been made to understand the importance of more exotic phenomena like π-π stacking in crystal nucleation and habit, where hybrid modelling – experimental approaches have been employed (102,103). The α polymorph of para-amino benzoic acid (α-PABA) was picked as the model compound because its structure gives rise to π-π stacking. The results suggest that the nucleation is governed by hydrogen bonding, whilst π-π stacking governs crystal growth, therefore determining the crystal habit. The authors claim that the crystal growth of α-PABA is driven by a poly-nucleation roughening mechanism, in which solute molecules diffuse on the [01$\overline{1}$] facet and attach to it by means of π-π interactions. This mechanism appears to dominate the process independently of the supersaturation. Even though the exact nature of π-π stacking is still a matter of debate (104), one can argue that beyond π-π stacking, a number of other parameters could be involved in crystal growth, including the effect of different solvents at the interface with particular facets.

### The Influence of Additives in Crystal Growth

There is a solid framework for the prediction of crystal habits for pharmaceutical compounds, from first principles, for different solvents and supersaturations. However, for the robust design of pharmaceutical formulations, it is important to understand crystal growth in environments containing many solute additives (105–107); additives influence crystal growth, interacting with individual crystal facets. It has also been shown that the majority of impurities incorporated into the crystal lattice are solute molecules and not solvent inclusions (108). The key to understanding the influence of additives in crystal growth, is to appreciate that the additive molecules interact anisotropically with different facets, competing with the molecules of the solvent and the other solutes. Thus, they can adsorb on specific facets, limiting their growth.

Chernov (47), Cabrera (109), and Vermilyea (110) have extensively studied the influence of impurities on the development of surface dislocations on crystals and therefore crystal growth. Those studies set the groundwork for the development of a mechanistic framework (111,112) for the prediction of the influence of additives in crystal growth in the context of the infamous BCF model, mentioned earlier. It has been shown that additives posing similar structure with the molecules of the crystal can be used as effective crystal habit modifiers. The extent of their influence appears to be a function of the additive concentration. Molecules of pharmaceutical interest have been employed in these studies to verify the importance of structural similarity; glycine was grown in the presence of another amino acid, alanine, and acetaminophen was grown in the presence of p-acetoxyacetanilide (113). For the amino acids, alanine shows an affinity to the [020] facet, whereas p-acetoxyacetanilide seems to be attracted to the (109) facet of acetaminophen. Furthermore, using similar mechanistic approaches, the results obtained from the combination of MD and the Wulff-Chernov formalism, in the context of the earlier urea-biuret system, have been verified (113–115).

Modelling attempts, grounded on the mechanistic models discussed in the previous section have been developed. Interestingly, urea was one of the molecules of interest. MD simulations, verified that the biuret additives interact anisotropically with different facets (57). The results were again implemented in the modelling framework which managed to predict accurately the crystal habit in the presence of biuret.

This concept, of structurally similar additives as crystal growth inhibitors, has been successfully implemented for the development of therapeutic approaches against kidney stones (116–120). A combination of experimental, modelling, and theoretical studies were employed to select the most appropriate molecular imposters from a library of candidate compounds. The growth velocities of the individual facets have been investigated with the aid of AFM. Interestingly, a case of crystal growth acceleration owing to the presence of additives was reported in this study. Comparable results have been reported for the growth of l-alanine in the presence of l-valine. The acceleration has been attributed to the enhanced solvation of the crystal by water because of the strongly hydrophobic nature of the additive (121).

Similar approaches have also been used to tackle malaria (122–124). The structure of hemozoin, the inert crystal that malaria parasites crystallize in their digestive vacuole to accommodate for the toxicity of heme, crystals was resolved enabling the determination of the facet specific attachment energies. In this context, it was possible to develop a rationale for understanding the action of quinoline based drugs. More in depth studies lead to the development of screening platforms (125) for the selection of optimum drug candidates based on growth inhibitor activity (115–117).

The hitherto presented approaches are based on the use of small molecule additives. However, it has been demonstrated that polymeric and surfactant additives can influence crystal growth (126–129). Going a step further, Rimer *et al*. (52,118) studied the influence of ionic polymers in the inhibition of kidney stones. In these studies, it was highlighted that polyanions were interacting much better with crystal surfaces compared to polycations. Furthermore, it was shown that beyond crystal growth, polymeric additives contribute to the aggregation of crystals. In fact, it was demonstrated that in mixtures of polyanions with polycations, the protein aggregates formed were mediating the formation of crystal aggregates.

The use of seed crystals is a key method used for the control of crystallization processes industrially (130–132). From the hitherto analysis, and from an intuitive understanding of crystal growth mechanisms, it is obvious that as long as crystal growth is allowed to reach equilibrium, the crystal habit of the seeds does not influence the crystal habit of the final product. This concept has been proved experimentally, using succinic acid as the model compound. It was shown that for single crystal growth, the equilibrium crystal habit is independent of the seed’s habit (42). However, as the concept of seeding is expanded in the field of industrial crystallization, precautions should be taken in order to minimize the influence of homogeneous nucleation. Excessive homogeneous nucleation would shift the heterogeneous nucleation controlled system to a homogeneous nucleation controlled one. This would eliminate any potential advantages offered by seeding (133).

#### Factors Affecting Crystal Dissolution

Different physicochemical characteristics of crystalline solids can influence the dissolution rate. These characteristics include crystal defects, habit, surface area to volume ratio, surface chemistry, surface energetics, and physiochemical stability (polymorphism and the propensity of forming hydrates or solvates) to name a few, all of which were covered in the fundamental section of the review. The influence of these material characteristics on dissolution behaviour often overlap. The following sections will seek to isolate and develop these points from the perspective of their effect on crystal dissolution.

### Anisotropy and Wetting of Crystalline Solids

As stated earlier, surface wetting is a prerequisite for the dissolution of materials. The process of surface wettability can be quantified in terms of fundamental concepts of work of adhesion and surface energy. Hence for the purpose of simplicity, some of the studies discussed here are from the perspective of wettability changes and involve surface energetics.

The surface energy of crystalline pharmaceutical materials, as has been shown throughout this review, is anisotropic (4). Considering facet specific surface energy of a crystalline material, it is postulated that surface energetics of the bulk crystalline material depends on the relative surface energy contributions of different crystal facets. For acetaminophen, Heng *et al*. reported that when milled, the dispersive component of surface energy increases with decreasing particle size and it was attributed to the surface energy of the weakest attachment energy plane for the acetaminophen crystals (3). Along similar lines, different studies have reported the facet specific wetting behaviour due to surface energy anisotropy of different crystalline facets. Lippold and Ohm reported that the surface wettability is correlated to the dissolution rate of solids through the effective surface area in contact with liquid at constant experimental conditions, notably constant agitation speeds. The wettability of the drugs was assessed by measuring contact angles with isopropanol –water mixtures and surfactant solutions (134). In the studies performed by Modi *et al*. (135,136), using celecoxib as the model compound, the influence of crystal anisotropy in dissolution was assessed and it was found that systems that exhibited more facets with higher surface energies showed greater bioavailability.

Despite the promising and reasonable conclusions, one should note that the contact angle measurements employed in the studies by Modi and Lippold were on powder compacts. During compaction, process breakages and defects arise on the crystals, as can be seen from relevant SEM images. It should be noted that the extent of the influence of these mechanically induced features are themselves heavily influenced by the crystal habit, meaning that different crystal habits will be modified differently under identical compaction conditions. This phenomenon, stemming from the concept of anisotropic mechanical properties of crystalline materials, casts doubts on the accuracy of the correlations between the contact angle surface energy measurements and wettability. From a theoretical perspective, it would be better for contact angle measurements on powder compacts to include notions based on the Wenzel and Cassie-Baxter eqs. (137–139).

### Crystalline Defects and Dissolution

The mechanisms underpinning the influence of defects on dissolution are multifaceted. Considering that defects, depending on their type, can influence both surface and bulk properties, one could expect that obtaining correlations for the influence of defects is not a straightforward process. In the absence of any defects, it is expected that crystal habit greatly determines dissolution; however, investigators have shown that this was not the case for acetaminophen. In fact, the dissolution rate trend for the facets was not in line with the attachment energy trend (140) used in the Hartman and Perdok predictive model. Thus, X-ray topography was employed to investigate the effect of defect induced strain on the crystal. Acetaminophen crystals with two distinct crystal habits were used: columnar crystals, obtained at low supersaturations, and prismatic shaped crystals, at elevated ones. The results suggest that the abundance of defects incorporated within the crystal lattice create a strain which enhances dissolution. They did not suggest a primary mechanism of defect formation, though, as discussed earlier in the review, we know that the inclusion of defects is an integral part of crystal growth. The effect of defects induced solely by mechanical processing is more often discussed since it appears to be more industrially relevant.

Particular attention is given to milling, a process used extensively downstream of crystallization. It typically induces disorder into the crystal lattice, which varies depending on the material. DSC and PXRD are not always adequate to quantify this disorder, and solution calorimetry (141,142) has been employed as a more accurate quantification method. Shah *et al*. investigated the behaviour of recrystallized brivanib alaninate, milled at both cryogenic and ambient temperatures (143,144). It was found that milling at cryogenic temperatures lead to higher disorder upon milling. This phenomenon suggests that the mechanical properties of crystals change in cryogenic temperatures, leading to more brittle structures. It should be noted that extensive mechanical processing can lead to partial amorphization of the material, especially on the vicinity of the surface. The lack of long range order, associated with amorphous materials, leads to higher reactivity. Thus, wettability, the first step of dissolution, becomes faster.

### Effect of Polymorphism on Dissolution

Polymorphism in crystalline solids and its effect on the performance of pharmaceutical products, has been the subject of extensive research over several years. The ability of a molecule to exist in different crystal forms influences the saturation solubility (C_s_) parameter affecting the dissolution rate; it can drastically alter the dissolution behaviour of an API or the formulation and its bioavailability (145,146).

A well-known industrial case study for an unexpected and unwanted polymorphic transformation was that of Ritonavir, an antiretroviral medication. After a couple of years of its commercialization, a new and previously unknown metastable polymorph was discovered, with poorer bioavailability than the corresponding original crystalline form (147). In a way, such case studies showcase the importance of polymorphic transformations in crystalline solids as related to therapeutic efficacy. Metastable polymorphs can be used to enhance the dissolution rate; however, enhanced dissolution with such polymorphs does not always translate into improved bioavailability (148).

The anhydrous form of a crystalline solid is generally more aqueous soluble than the so called pseudopolymorphic hydrate, such as in carbamazepine dihydrate from earlier. Therefore, hydrate formation can influence the solubility and the dissolution of solids. This could be a substantial issue in the case of moisture sensitive substances where the transformation from anhydrous to hydrous form occurs with the uptake of moisture from the environment. Bartolomei *et al*. documented a decrease in the intrinsic dissolution rate of hydrated diclofenac sodium, formed on exposure to humid conditions, than that of the corresponding anhydrous diclofenac sodium salt. Moreover, the transformation of metastable polymorph to its stable form may occur during the dissolution process. Often this is influenced by the dissolution medium as suggested by several studies on solvent mediated solid-state transformation of certain APIs (149–153). An interesting study by Lehto *et al*. reports the complex dissolution behaviour of molecules susceptible to hydrate formation, by studying simultaneous dissolution and crystal growth processes of carbamazepine form III and carbamazepine dihydrate respectively using *in situ* analytical techniques. They reported solvent mediated crystal habit changes of the carbamazepine dihydrate crystals in the dissolution media of normal simulated intestinal fluid (SIF) and SIF containing surfactants, and the corresponding changes in the dissolution kinetics. These crystal habit changes were attributed to hydrogen bonding effects between the carbamazepine dihydrate and surfactant molecules.

## Crystal Engineering for FAVOURABLE Dissolution

Poor water solubility is evidently linked to poor dissolution which could consequently result in worsening bioavailability. Therefore, extensive efforts and resources are being invested in both the solubility and dissolution enhancements of drugs. It is important to mention that this section deals with approaches strictly pertaining to the engineering of crystals for dissolution improvement, and omits literature studies where formulation factors are responsible in improving dissolution. Both bottom up (during crystal growth), and top down (processing of crystalline materials, or nano/micro- crystallization) approaches can be used in conjunction with cocrystallization.

### Crystal Habit Modification

The significance of surface area in dissolution kinetics, makes crystal habit and crystal size critical factors during the dissolution process. Solvents, crystal growth inhibitors, and additives (154,155), as discussed earlier in the review (20), have been proven to be particularly successful at controlling crystal dissolution rates (156).

Although variable dissolution rate changes, due to crystal habit, can be correlated to surface area, sometimes the physicochemical changes in surface characteristics, such as defects and surface chemistry, may contribute to such behaviour. Adhyayiman and Basu reported the improvement in the dissolution rates of dipyridamole crystals, based on their crystal habits. Two different crystallization setups were used. The first involved the use of different solvents, whereas the second used additives such as Tween-80, PEG, and PVP (157). It was previously reported that the relative exposure of different crystal facets is related to the degree of wettability, and subsequent dissolution, of an API (158). Modi *et al*. studied the influence of plate and acicular crystals of celecoxib on their intrinsic dissolution rates (IDR). The IDR of the plates were about 50% greater than that of acicular ones in a phosphate buffer. The greater wettability of the plates, as compared to acicular shaped crystals, was found to be responsible for the variation (135). Sometimes a combination of factors, such as surface area and surface chemistry, can improve the dissolution rate of the crystals. Chow *et al*. reported an increase in the IDR of doped phenytoin crystals, where the habit was controlled, by varying the specific surface area alongside the increased density of surface polar groups at the facets (159).

Dissolution is an inherently dynamic process during which the size of the crystal decreases along with its habit. The process is driven by the preferential removal of lattice components from the edges of the crystal. Inevitably, this leads to the appearance of new facets, as the crystal shrinks from the edges. In other words, the crystal becomes smaller but more heterogeneous, exposing more facets. This is different to crystal growth where the fastest growing facets tend to disappear so less are exhibited (160). However, experimental studies, performed in the absence of convective mass transfer, show that as the dissolution process progresses, crystals take a more rounded shape (161). This suggests the presence of many more crystal facets being exhibited than as predicted from simulations. This behaviour has been attributed to the effects of local roughening on each facet. This roughening is associated with the growth mechanisms brought forth by Burton, Cabrera, and Frank; however, their effects are often omitted in favour of better simulation performances. In experiments performed in the presence of some sort of stirring, this rounding phenomenon occurs for larger crystals than when observed in purely diffusional experiments. The shear stress associated with stirring further enhances the formation of local defects and roughening on individual facets, promoting this rounding effect.

### Post-Crystallization Approaches

Owing to the heterogeneous nature of crystalline materials, researchers are exploring numerous strategies to tailor particle surfaces for controlling properties such as surface wettability and solubility. Since the physicochemical nature of surfaces is critical in determining these properties, the emphasis remains on the development of technologies which can ensure that changes occur only at the particle surface, keeping the particle bulk properties intact. This section deals with the post-crystallization treatments of surfaces to produce tailored crystalline materials to improve dissolution properties (162).

### Surface Modification

Gaining control over surface chemistry appears to be the preferred method for controlling the wetting and dissolution characteristics of pharmaceutical materials. Surface functionalized coating of pharmaceuticals is the most popular technique employed in the industry to modify the dissolution profile of APIs. Functional polymeric coatings from solvents are the most commonly used approach to obtain APIs with desirable dissolution properties (163). Profiles for the latter may include increased dissolution rates as well as controlled release systems (164). Moreover in the future, applications of potential surface functionalization techniques, like surface silanization, could be explored to modify the surface chemistry (143) and wettability, to alter the dissolution behaviour of crystalline materials.

**Fig. 6:**
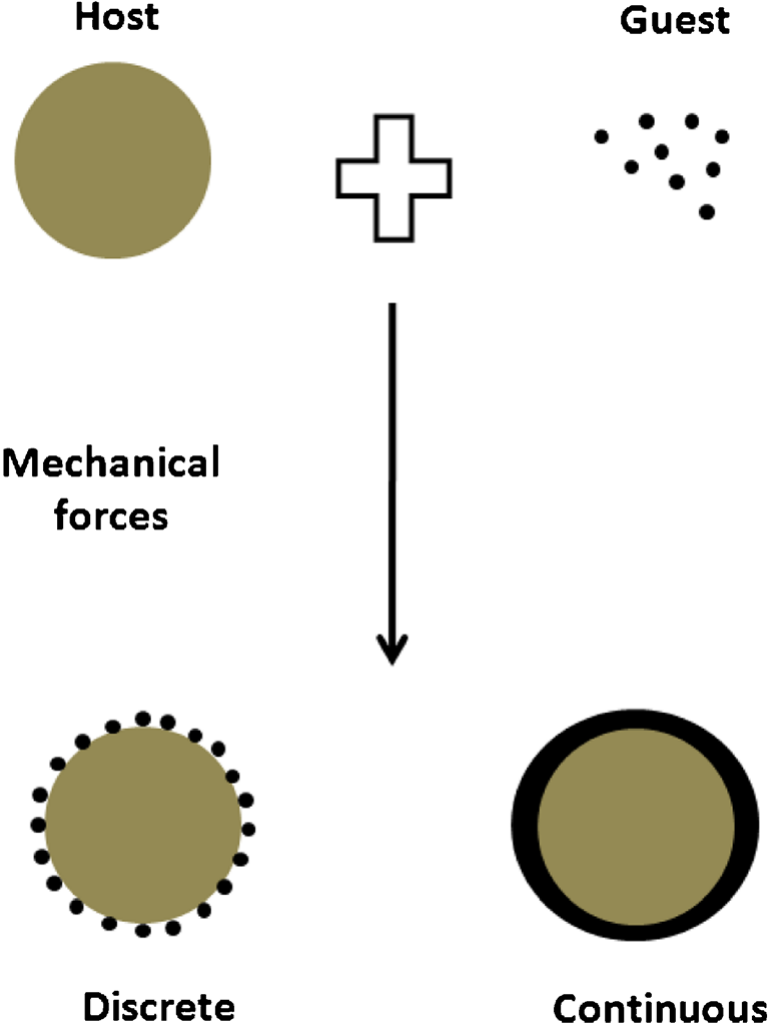
Schematic of dry particle coating

Apart from solvent based methods, dry coating methods have, also, been used by various researchers for particle surface modification to improve dissolution (165,166), wettability, as well as powder properties. In this technique, the coating of guest (fine) particles over host (coarse) particles is achieved by mechanical forces as depicted in Fig. 6. Recently, Han *et al*. reported a simultaneous micronization and surface modification technique for improving the dissolution of ibuprofen. This was achieved by co-grinding the drug with a water soluble hydrophilic polymer in a continuous fluid energy mill (FEM) to obtain surface modified particles. The dissolution rate enhancement in ibuprofen was ascribed to the resulting improvement in the wettability of the dry coated API (165). Generally, with proper operation control, co-grinding allows for enhanced dissolution without changing the crystalline form of the drug (166,167). In another similar study, Tay *et al*. performed the mechanofusion of poorly water-soluble indomethacin with MgSt and NaSt. An increase in the dissolution rate of the NaSt coated API, attributed to the combination of its ability to act as a surfactant and both promote drug wettability and drug dispersion in the dissolution medium, was observed. The dissolution profile was modelled using a non-linear least squares regression analysis with a bi-exponential eq. (166). Furthermore, Karde and Ghoroi showed that depending on the nature of the guest particle (hydrophilic and hydrophobic) employed for coating, modulation in the wetting behaviour of host surfaces is possible. Thus, dry coating using silica nano-particles (guest) with diverse functionalities led to wettability variation in several pharmaceutical excipient (host) surfaces (168). Largely, such solventless dry particle coating technique offer several advantages over the conventional wet or solvent based techniques for surface modification and dissolution enhancement of powders.

### Co-Crystallization

One crystal engineering approach, which has become popular these past few decades, for improving the solubility, dissolution, and bioavailability of drugs is co-crystal formation. Its popularity as a pharmaceutical dosage form design strategy is obvious by the numerous reviews and studies on the topic, as well as an ever-growing number of patent applications (156,169–174). A co-crystal can be defined as a crystalline material that consists of different molecular species held together by hydrogen bonds or non-covalent forces (175). Sometimes the terms ‘co-crystal’ and ‘molecular complexes’ are used interchangeably (176) to describe the hydrogen bonded molecular arrangements between two separate molecular entities, however some may disagree (177). Interestingly, one of the earliest studies on co-crystallization was by Ito and Sekiguchi (178), focusing on two sulphonamide drugs (sulfathiozole and sulphanilamide), reported this mixture as a ‘molecular compound’. These unclear terminologies for describing co-crystals have given rise to some other conflicting views whereby solvates, hydrates, and inclusion complexes (179) are sometimes considered co-crystals.

Pharmaceutical co-crystals can improve bioavailability of poorly water soluble drugs by enhancing the solubility (180) and dissolution (181–183) of the crystalline APIs. Several studies have investigated the intrinsic dissolution rates of co-crystals. Lee *et al*. investigated the dissolution characteristics of an acetaminophen/theophylline (AT) co-crystal (184). Higher dissolution rates for various AT co-crystals compared to their individual components and physical mixtures, were observed. In another instance the IDR of a poorly soluble API was drastically increased by a factor of 18 via co-crystallization with coformer glutaric acid (182).

Due to their superior physicochemical properties (185), co-crystals provide a suitable alternative to salts, hydrates, solvates, and polymorphs of APIs in the development of dosage form, thus circumventing some of the limitations associated with the latter compounds. For pharmaceutical applications, co-crystals can be formed between the active and a non-active co-crystallizing agent (co-former) (179,183), or between two active ones to combine their therapeutic effects (186). Crystal packing structures and lattice energies should be considered during co-crystallization. Molecules that can arrange themselves in alternative packing patterns whilst forming hydrogen bond networks are preferred molecules for co-crystallization [176]. Thus, finding polymorphs which are not energetically locked into single packing modes and exhibit structural flexibility, are good candidates for co-crystal formation (175).

The two most popularly used methods for preparing co-crystals include the evaporation of a heteromeric solution, or co-grinding the components together, called neat grinding (187,188). Caira *et al*. prepared co-crystals of sulphamidine with aromatic carboxylic acids using neat grinding. From their study on the preference for co-crystal formation (competitive experiments), they inferred that it is favoured by the decreased intermolecular interactions in homomer crystals as compared to the heteromers. As an alternative to the neat co-grinding process for co-crystallization, Jones and co-workers suggested a process called liquid-assisted grinding (LAG), in which the addition of small amounts of a liquid can dramatically increase the co-crystallization yield. However, in some cases the use of solvents prompted the formation of solvates. In subsequent work, it was claimed that greater control of the outcome could be achieved by using a solvent of appropriate polarity (189). This was further corroborated by Fischer *et al*. (190) with their work on theophylline and benzamide co-crystallization using LAG over a range of solvents. To avoid the possibility of solvate formation during co-crystallization, Jones *et al*. recently introduced polymer assisted grinding (POLAG) that provides advantages comparable to the conventional liquid-assisted process along with better control over the particle size of co-crystals (191).

Apart from the above mentioned approaches for crystal engineering to enable better dissolution, a popular top-down approach of micronization through milling, is widely employed in the pharmaceutical industry to improve the dissolution of crystalline material (192). The reduction in particle size and consequentially the increase in surface area due to micronization is thought to be main factor responsible for dissolution improvement. As discussed in the earlier sections of this review, mechanically induced defects generation in crystalline solids also play a crucial role in this process. However, it is worth mentioning that co-crystals are not always more soluble, i.e. dissolve in greater amounts than their corresponding coformers. This could happen because of the rapid precipitation of the poorly soluble drug, which could form a layer of precipitate on the soluble co-crystal and thus affect the formers dissolution. Recently, Yamashita and Sun showed that this precipitation phenomenon, of the low soluble entity of co-crystals, could be reduced by adding excess coformer to the dissolution medium, which they have named the ‘common coformer effect’ (193,194).

## Comments and Perspectives

### Crystal Growth and Dissolution Are Not Reversible

This review highlights the importance of crystal habit in both crystal growth and dissolution in solution. These two processes, of crucial importance in the pharmaceutical industry, are inherently different and they should not be approached as opposites nor reversible. It is crucial to understand that the underlying mechanisms for these two processes are unique as has been highlighted in this review.

Dissolution is initiated by the wetting of a solid by the undersaturated bulk fluid. As long as the wettability is established, a diffusion layer is formed. The rate of dissolution is then established, where the process is governed by the size of the diffusion layer, the magnitude of the diffusion coefficient, and the concentration gradient between the bulk and the surface. The different surface chemistries of individual facets leads to anisotropic wettability, changing the time needed for the dissolution to be initiated on each facet. Similarly, the rate of detachment of molecules from the surface of the crystal during dissolution is also facet specific.

On the other hand, crystal growth takes place in a supersaturated solution. It is well established that the degree of supersaturation is one of the key parameters driving it. However, interfacial phenomena, linked with crystal habit, are also of crucial importance. The concept of critical step length, as mentioned earlier, is the ratio linking interfacial to bulk phenomena. The solid-liquid interfacial (γ_SL_) tension, on the numerator of the equation describing critical step length, is facet dependent. In the case of strong affinity between the solvent and the facet, the magnitude of γ_SL_ is large leading to a large critical step length. The bigger it is, the greater the force limiting the influence of supersaturation in crystal growth (195). In other words, if the liquid and the solid have a strong affinity, more energy is required to form a new solid surface area. Hence, wettability counterbalances the effects of supersaturation.

Unfortunately, there is a dearth of studies in the literature discussing the importance of the different components of surface energy in crystal growth. From a theoretical perspective, the step size should vary for different solvents and different values of pH. In any case, a strong understanding of the interactions, both electrostatic and specific, associated with individual facets is required. However, for a wide range of materials, including proteins, contact angle goniometry and XPS measurements as those proposed in the literature (4,5,7) are not experimentally feasible, where similar issues limit the applicability of IGC (41). Chemical force microscopy (CFM), could be a promising tool (196–199) as it enables the measurement of the interactions of a specific facet with a functionalized AFM tip. Combining many functionalization groups against a specific facet, could provide a good understanding of the interactions associated with that facet. The Quartz crystal microbalance (QCM), may provide a useful platform to study facet specific interactions with different functional groups. In this direction, some progress has, already, been achieved in the use of QCM to study crystal growth (200,201).

It should be appreciated that during crystal growth, especially at low supersturations, there is an interplay between attachment and detachment frequencies of molecules on different facets. Molecules can attach or detach from the crystal facets, though because of the nature of the process, the former will dominate the latter as is evident in all the MD studies.

### The Importance of Surface Active Additives in Growth and Dissolution

The presence of additives in solution, giving rise to surface activity, changes its wettability. In the presence of surface active agents such as polymers (202,203), and surfactants (204,205), the wettability mechanism is driven by their tendency to migrate towards the three phase contact line (69,70). This influence has been highlighted in a very detailed way in the concept of carbamazepine dihydrate (129). The interactions of the hydrophilic components of sodium taurocholate with the (110) facet of the carbamazepine dihydrate, via hydrogen bonds, limits the growth of that facet, leading to crystals with smaller aspect ratios. Similar studies were conducted with aspirin (206) and nifedipine (89,207,208).

Similar arguments apply for the importance of specific surface interactions with additives in the dissolution process. The influence of a wide range of polymers in the dissolution of crystalline pharmaceutical materials has been investigated. It is evident that there is a lag time between the contact of a tablet with a solvent and the start of drug release (209–212). This lag phase is influenced by the wetting mechanism of the drug by the surrounding solvent. Higher wettability, linked with lower surface tension of the liquid surrounding the drug, would decrease this lag phase. Increasing the amount of polymer used and its physicochemical properties alters the surface tension and therefore the lag time. One should be careful when performing or assessing such experiments with tablets, since residual powder on the surface of the tablet should be removed, as it dissolves quite fast and thus makes the lag phase difficult to be identified. This lag behaviour can also be seen in amorphous formulations.

The interpretation of the impact of additives on the components of the surface tension, the Lifshitz-van der Waals and the acid-base ones, remains unclear. It is not unreasonable to speculate that the solubility parameters could provide a metric enabling us to quantify the impact of additives by these individual components. Further studies should also be conducted to understand the changes in the solvent properties by the addition of additives.

### Particle Sizing Is Crucial

The reader should appreciate that when considering the size of the particles used in drug formulation, usually in the micron and submicron scale, interfacial phenomena tend to be of increasing importance. One should also note that there is a tendency towards the introduction of smaller particles in pharmaceutical formulations (213,214). This tendency for smaller particles will, therefore shift the balance towards interfacial phenomena. The advantages of using particles in the nanometre scale are extensive and many of them can be easily conceptualized in terms of the fundamental interfacial phenomena outlined in the theoretical development part of this work.

Working at the nanoscale poses challenges for researchers. Therefore, it is of crucial importance to further the development of bottom-up and top-down techniques for the synthesis of nanoparticles with specific engineered properties. Especially for top-down approaches, the importance of crystal habit and crystal anisotropy in milling and breakage cannot be understated. The production of smaller particles should be accompanied by the development of theoretical, computational and experimental approaches for the understanding of interfacial phenomena at the nanoscale. This should go beyond the well-established concepts of Ostwald-Freundlich theory. It could focus on the influence of the decrease in size in the lattice properties giving rise to surface energy. Finally, the implementation of nanoparticles in process development and their storage could be challenging, considering the variety of undesired phenomena associated with them, including coalescence upon storage and extensive cohesiveness.

In addition, traditional pharmaceutical unit operations, like spray drying, can be used to modify the microstructure of crystalline materials. A study published by Rasenack *et al*. demonstrated that the dissolution enhancement of poorly water-soluble drugs could be achieved by the formation of microcrystals using precipitation techniques and spray drying. The crystals could be produced with high specific surface areas and higher surface hydrophilicity with stabilising protective polymer facilitating dissolution (215).

### Conclusions

Crystal growth and dissolution remain key phenomena in pharmaceutical process development and they will probably retain this status for the foreseeable future. Their study requires a strong understanding of the interplay between surface and bulk properties of materials. The focus of this work has been on interfacial phenomena and crystal anisotropy. It is evident that the combined use of theoretical, computational and experimental approaches is paramount to the development of predictive tools for crystal growth and dissolution.

The shift towards sub-micron/nano particles, for the enhancement of bioavailability, makes the importance of interfacial phenomena more prominent in the field of pharmaceutics. Interfacial phenomena can be exploited for the development of strategies for the improvement of the rate of dissolution of crystalline materials. However, it is becoming evident, that there are still big discrepancies associated with the quantitative understanding of interactions at the solid-liquid interface.

## Abbreviations

A: Unit surface area (m^2^)
AFM: Atomic force microscopy
BFDH: Bravais-Friedel-Donnay-Harker
BCF: Burton-Cabrera-Frank
C: Concentration (mol/m^3^)
C_0_: Initial concentration (mol/m^3^)
C_b_: Bulk concentration (mol/m^3^)
C_s_: Surface concentration (mol/m^3^)
CFM: Chemical force microscopy
D: Diffusion coefficient (m^2^/s)
d_hkl_: Interplanar spacing (m)
DSC: Differential scanning calorimetry
${E}_{hkl}^{AE}$: Bond attachment energy (J/mol)
H: Boundary layer thickness (m)
h_s_: Step height (m)
IGC: Inverse gas chromatography
IR: Infrared
J: Mass flux (mol/m^2^s)
K: Dissolution rate constant (m^3^/s)
L_c_: Critical length (m)
M: Mass released (kg)
MD: Molecular dynamics
NMR: Nuclear magnetic resonance
Q: Solid mass (kg)
R_hkl_: Growth rate of facet hkl (m/s)
SAFT: Statistical association fluid theory
T: Time (s)
V: Step velocity (m/s)
vOCG: van Oss-Chaudhury-Good
W_12_: Work of adhesion between surfaces 1 and 2 (mJ/m^2^)
X: Direction vector (m)
XRD: X-ray diffractometry
XPS: X-ray photoelectron spectroscopy
Y: Interstep distance (m)

## Greek Letters

γ^Total^: Total surface energy (mJ/m^2^).
γ^LW^: van der Waals surface energy (mJ/m^2^).
γ^AB^: Acid-base surface energy (mJ/m^2^).
γ^+^: Acid component of surface energy (mJ/m^2^).
γ^−^: Alkaline component of surface energy (mJ/m^2^).
γ_SL_: Solid-liquid interfacial energy (mJ/m^2^).
γ_hkl_: Surface energy of facet hkl (mJ/m^2^).
Δμ: Chemical potential difference (J/mol).
$\Delta {G}_{hkl}^{\ast }$: Activation free energy for the incorporation of a solute molecule on the facet hkl (J/mol).
